# Clinical Society of Baltimore

**Published:** 1893-11

**Authors:** 


AT the 29th regular meeting of the Clinical Society of Baltimore, Md., the following officers were elected for the ensueing year: President, Dr. J. Edwin Michael; Vice-President, Dr. Hubart Horlan; Rec. Sec., Dr. H. O. Reik; Corres. Sec., Dr. W. T. Watson; Treasurer, Dr. W. J. Todd; Executive Committee, Dr. J. M. Hundly, Dr. W. T. Lockwood and Dr. J. M. Craighill; Member Finance Com., Dr. W. Green.

